# Management of a Buccal Space Mass: A Clinical Case Report

**DOI:** 10.1155/2020/6828453

**Published:** 2020-12-14

**Authors:** Alexander Karatzanis, Stylianos Velegrakis, Georgia Liva, Dionysios Kyrmizakis, Emmanuel Prokopakis

**Affiliations:** ^1^Department of Otorhinolaryngology,Head and Neck Surgery, University of Crete Medical School, Giofirakia, Greece; ^2^Department of Otorhinolaryngology,Head and Neck Surgery, General Hospital of Veroia, Papagou, Greece

## Abstract

**Background:**

Buccal space tumors constitute rare pathologies with significant histological diversity. They may pose serious diagnostic and therapeutic challenges for the head and neck surgeon.

**Methods:**

A case of buccal space tumor diagnosed and treated in a tertiary center is presented. Clinical presentation, imaging, and surgical approach are discussed, followed by review of the literature.

**Results:**

A 79-year-old male patient with a slowly growing painless mass on the right cheek presented to a head and neck reference center. Imaging revealed a tumor of the right buccal space with nonspecific characteristics. Imaging studies revealed extended infiltration of the masseter muscle as well as the anterior border of the parotid gland. FNA biopsy was performed but was nondiagnostic. The decision of surgical excision with a modified parotidectomy incision was taken. The lesion was completely excised with preservation of neighboring facial nerve branches and ipsilateral Stensen's duct. The postoperative course was uneventful. Histological examination showed CLL/Lymphoma, and the patient was referred to the hematology department for staging and further management.

**Conclusion:**

Differential diagnosis of buccal space masses is very diverse. Despite challenges in the diagnostic and therapeutic approach, these entities may be managed surgically with minimal morbidity.

## 1. Introduction

Buccal space pathologies are relatively rare and thus seldom encountered in the literature. Despite occult epidemiology, buccal space masses show significant clinical and etiologic diversity [1, 2]. In addition, the complexity of buccal space anatomy and relative aesthetic considerations all contribute to the significant diagnostic and therapeutic challenges that buccal space masses pose for the head and neck surgeon. In this report, a rare buccal space tumor is presented, diagnosed, and treated in a head and neck reference center. Furthermore, a detailed description of the anatomy and review of the literature are provided, in an attempt to shed light to all clouded areas regarding epidemiology, diagnosis, and management of buccal space masses.

## 2. Case Report

**Figure 1:**
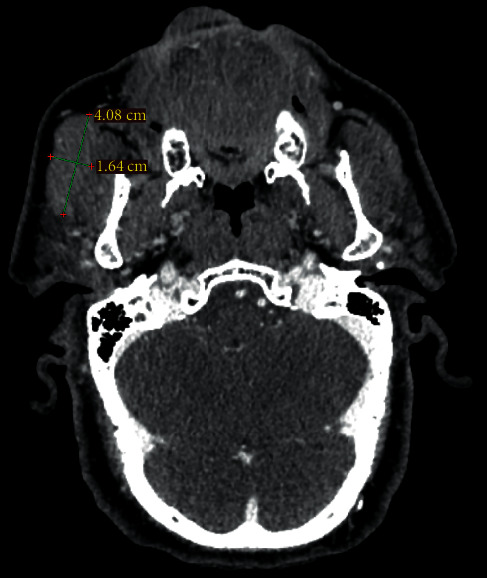
Computed tomography showing a buccal space mass with irregular margins and invasion of the neighboring masseter muscle.

**Figure 2:**
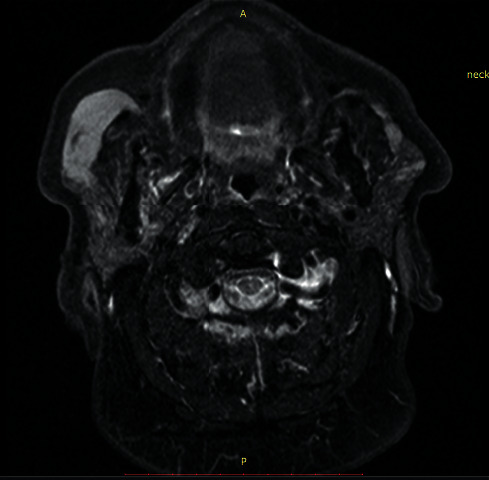
MR T2 sequence showing a buccal space mass.

**Figure 3:**
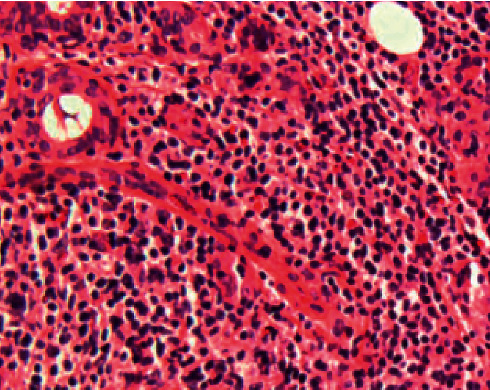
Lymphocytic proliferation of the parotid gland (hematoxylin and eosin).

**Figure 4:**
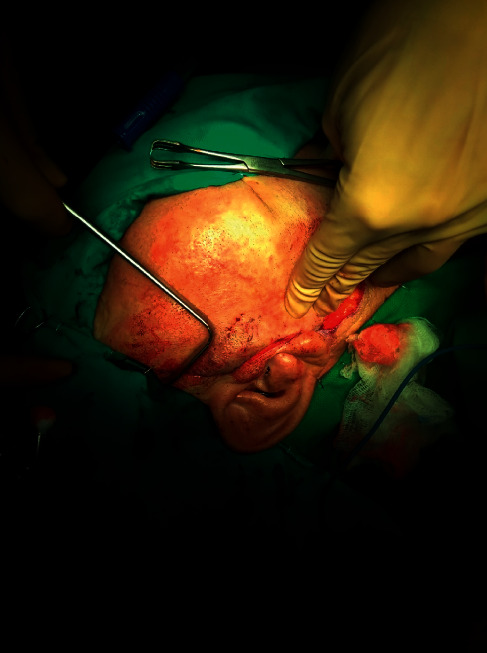
Extended parotidectomy incision for the surgical excision of the buccal space mass.

**Figure 5:**
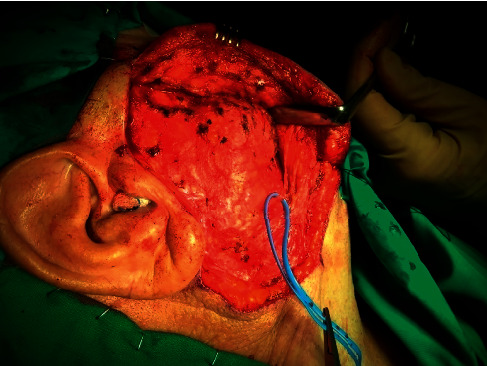
Preparation of a subplatysmal flap for good exposure of the mass. Neighboring branches of the facial nerve are identified and preserved.

**Figure 6:**
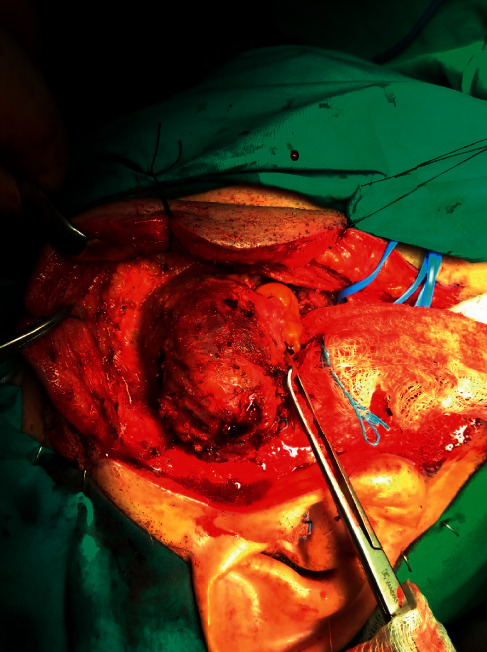
Careful dissection is performed, and the mass is followed anteriorly into the buccal fat pad.

**Figure 7:**
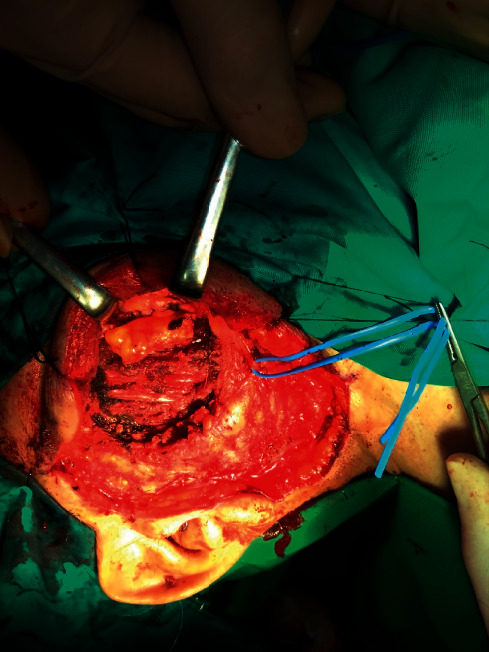
Surgical field following excision of the mass. A large portion of the masseter muscle has been removed en block with the tumor.

**Figure 8:**
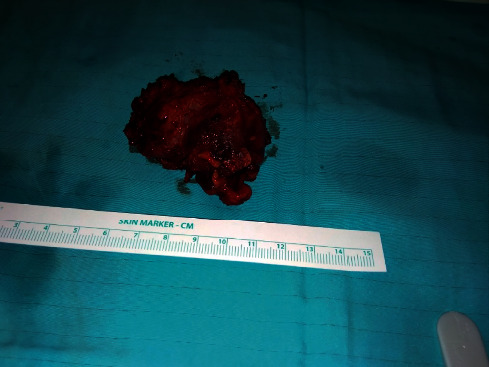
Surgical specimen following excision.

A 79-year-old male patient presented with a painless tumor of the right cheek, located below and laterally to the right zygomatic bone. The mass had existed for at least six months and was slowly increasing in size. Hypertension, diabetes, hyperuricemia, and hypercholesterolemia were mentioned in the medical history. No previous significant pathology or surgery was reported in the head and neck area. Computed and magnetic tomography, with 1.25 mm thickness cuts in the transverse plane and with image reconstruction cuts of 1.25 and 2.5 mm in coronal and sagittal planes, respectively, revealed a lesion of 1.64 cm × 4.8 cm in dimensions which occupied the right buccal space, originating from the right major zygomaticus muscle, and extending to the right anterior part of the parotid gland (Figures 1 and 2). There was homogeneity in composition with a distinguishable contrast intake in comparison with the healthy side and discrete vessels throughout the circumference of the lesion. The tumor had smooth outlined limits with projections in the anterior area of the right parotid gland and the lateral portion of the masseter muscle. A malignant lesion in the buccal space could not be excluded from the differential diagnosis. However, the characteristics of the lesion were not exactly compatible with typical malignancies of the area, including salivary gland carcinoma and lymphoma. Blood tests did not reveal any specific findings, with white blood test count on the lower normal levels. The patient did not present any B-type symptoms or showed any signs of localized lymphadenopathy. FNA biopsy was performed, retrieving numerous lymphocytes and nonspecific atypical cells, and was characterized as nondiagnostic (Figure 3). The patient refused to undergo a second FNA biopsy. The decision for excision under general anesthesia was then taken as the possibility of a malignant lesion could not be excluded. The patient underwent resection through a modified parotidectomy incision (Figures 4–8). The tumor was meticulously dissected and found to macroscopically infiltrate the anterior border of the parotid gland and deeply infiltrate the ipsilateral masseter muscle. Specific attention, with the aid of nerve monitoring, was taken for the identification and preservation of buccal and marginal branches of the facial nerve. A significant portion of the masseter muscle had to be excavated along with the tumor, and the specimen was eventually followed into the buccal fat pad and removed after careful identification of the parotid duct. Meticulous hemostasis and wound closure in layers with a closed-suction drain followed. Recovery from surgery was uneventful.

Permanent histology described a solid white/brown ulcerated neoplastic mass with a maximum diameter of 5.3 cm (Figure 3). Infiltration was found by a lymphohyperplastic neoplasm, with diffuse architecture containing small modified lymphocytes, prolymphocytes, and immunoblasts that built multiple proliferation centers. Immunohistochemistry was CD20+, PAX-5+, CD3−, CD5+, CD23+, CyclinD1, CD43+, CD10−, BCL6−, MUM1−, Ki67 15%, and Cd30 2%. These findings were compatible with chronic lymphocytic leukemia/lymphoma, and the patient was referred to the hematology department for further management. Staging was performed and revealed stage IVA B-lymphocyte lymphoma, with extralymph node spreads in the parotid area and thoracic paravertebral region (T5-T11) of the spinal cord, but without infiltration of the bone marrow. A management decision of watchful waiting was taken by the hematologists, and the patient was placed on close follow-up. One year later, the patient has remained clinically stable, and imaging findings are invariable.

## 3. Discussion

The buccal space is a small potential space on the lateral aspect of the face that forms the infrastructure of the cheek [1, 2]. Contents of the buccal space include adipose tissue, the parotid duct, facial artery and vein, minor salivary glands, lymphatic channels, and branches of facial and mandibular nerves [3]. Lack of complete fascial boundaries to three directions permits almost unimpeded spread of pathologic conditions to and from the buccal space [4, 5].

A variety of diseases may occur within the buccal space, including developmental lesions, inflammatory conditions, neoplastic lesions, either primary or metastatic, and miscellaneous diseases such as Kimura disease and foreign body granuloma [3, 6]. In approximately one-fifth of the general population, an accessory parotid gland is present in the buccal space, and it is histologically and physiologically identical to the main parotid gland tissue [7, 8]. Accessory parotid tissue may be unilateral or bilateral, is located superior to the parotid duct, and is lying on the masseter muscle anteriorly and separately from the main parotid gland [6]. The accessory parotid gland duct is emptied in Stensen's duct [7]. Pathologies which may lead to enlargement of the accessory parotid gland include sialadenitis, as well as benign and malignant tumors [1, 7, 8]. Among parotid neoplasms, accessory gland tumors occur at a rate between 1% and 8% [8].

Developmental lesions of the buccal space include dermoid cysts and hemangiomas [3]. Abscess formation may be possible when such lesions are complicated by infection. Apart from developmental pathologies, a wide range of inflammatory conditions may develop in the buccal space, owing to a variety of etiologies with sialadenitis being the most predominant. The obstruction of Stensen's duct, typically from salivary stones, may be the main causative factor of acute inflammation of the parotid area in adults.

The most common pathogens associated with acute viral parotitis are paramixovirus, adenoviruses, and rarely influenza A (H3N2) or coxsackie A or B [9–11]. Herpes virus is a rare cause of infection [11]. Acute bacterial parotitis may occur more often from Gram-positive pathogens such as *Staphylococcus aureus*, *Fusobacterium* spp., and *Streptococcus* spp [10–12]. Less frequent is parotitis from *Mycobacterium tuberculosis* usually in the context of extrapulmonary tuberculosis in an immunosuppressed patient [12, 13]. Finally, infections within the buccal space may develop on the ground of dental infection spreading typically from the masticator space. Many of the prementioned pathologies may be complicated by an abscess, fistula, or cellulitis [6, 10].

Neoplastic lesions of the buccal space include primary, benign or malignant, and metastatic. Salivary gland tumors, rhabdomyosarcoma, neurofibroma, and lymphoma constitute the majority of primary neoplastic lesions of the buccal space. Salivary gland tumors originate from the parotid gland in 80% of cases, and their epidemiology and clinical course are well established and beyond the scope of this report [14, 15]. Rhabdomyosarcomas constitute rare malignant mesenchymal tumors, which in approximately 36% involve the head and neck [3]. They extend to the buccal space from neighboring regions. Neurofibroma is a benign peripheral slow growing tumor, which rarely affects head and neck regions [3, 6]. Neurofibroma may occur as a solitary lesion or as a part of the generalized syndrome of neurofibromatosis (usually type-1) [6, 16]. Lymphomas constitute neoplastic malignant proliferation of the immune system and are divided into Hodgkin (which are predominantly B-cell neoplasms) and non-Hodgkin (which are either B-cell or T-cell neoplasms). Ten percent of lymphomas are Hodgkin and 90% non-Hodgkin [17]. Clinical presentation depends mainly on the lymphoma subtype. Non-Hodgkin lymphomas may appear with weight loss, night sweats and fever (B symptoms), or painless lymphadenopathy [17, 18]. Management and prognosis greatly depend on stage and patient performance status [17, 18]. However, up to one-third of non-Hodgkin lymphomas may be extranodal [19]. Non-Hodgkin lymphomas of the head and neck appear more often in men [17]. Primary non-Hodgkin lymphoma of the salivary glands is a rare condition accounting for about 5% of the extranodal non-Hodgkin lymphomas and 1.7% of the salivary gland malignancies [19, 20]. The salivary gland usually affected is the parotid, followed by the submandibular gland [19[[parms resize(1),pos(50,50),size(200,200),bgcol(156)]]reactive, benign, or malignant and to distinguish between salivary and nonsalivary lesions. However, because of heterogeneity of numerous lesions, the features of cytologic morphology are overlapping making the diagnosis extremely difficult [20, 21]. Therefore, patients usually undergo surgery in order for a definite diagnosis to be made [19].

Metastatic lesions in the buccal space occasionally derive from squamous cell skin carcinomas of the face and less frequently from lung, breast, and kidney cancer [3]. In rare cases, buccal space masses may occur from regional infiltration of paranasal sinus, maxillary bone, and nasopharyngeal carcinoma [22].

Kimura disease is a chronic inflammatory disorder of unknown etiology and is histopathologically characterized by lymph-folliculoid granuloma and pronounced eosinophilic infiltration [3, 23]. It presents as a painless lymphadenopathy or subcutaneous mass, particularly in the parotid and submandibular regions. Peripheral blood eosinophilia and an increase in serum IgE levels are typically present [23]. Finally, foreign body granuloma occurs from local injection of foreign materials, mainly for cosmetic reasons [3, 6].

Following clinical detection of a buccal space mass, detailed patient history and clinical examination are required. It is important to demonstrate location, depth, size, consistency, and mobility of the mass. Pain and signs of inflammation should also be noted. Apart from physical examination, an imaging study of the buccal region such as ultrasound, CT, or MRI will be needed. Ultrasound is used predominantly for superficial structures in order to distinguish solid from cystic lesions, gives information regarding vascular supply, and is able to detect enlarged regional lymph nodes [24, 25]. Ultrasonography provides excellent resolution and tissue characterization without radiation hazard [25]. Ultrasound-guided fine needle aspiration (FNA) biopsy may be performed to evaluate cytological examination of the mass for preoperative planning [24, 25]. For some clinicians, FNA biopsy should not be performed in order to protect branches of the facial nerve from injury [26]. In the present series, FNA biopsy was utilized with no complications and no diagnostic yield. MRI and CT may assess the complete tumor extent and the existence of regional lymphadenopathy. CT is the method of choice in cases suspicious for inflammatory disease (abscess, calculi, major salivary duct dilatation, and acute inflammation) or in patients with contraindication for MRI [24]. However, MRI is the preferred method for soft tissue imaging. It is very useful for assessment of tumor extension, marrow infiltration, and perineural spread and addressing relation with neighboring structures [25]. A decision not to proceed with an MRI was taken in the case presented here, as it would not alter the management decision of surgical exploration.

Branches of the facial nerve and the parotid duct are closely associated with tumors of the buccal space. Therefore, careful intraoperative mapping of the tumor and the neighboring regions is necessary. Several surgical techniques have been proposed at times. The incision of the ideal surgical approach should provide complete exposure of the surgical field, allow the conversion to radical surgery if it is needed, and produce a satisfactory cosmetic result [27]. Although the cosmetic result of the transoral approach is excellent, it lacks in popularity because neither complete exposure is provided nor expansion of the surgical approach is feasible when necessary [8, 27]. Furthermore, there is a great risk of facial nerve and parotid duct injury. Another controversial choice is a skin incision directly over the mass [8]. The branches of the facial nerve travel quite superficially as they leave the parotid gland and for this reason they may be injured. On the other hand, the extended parotid-submandibular incision fulfills most criteria for an ideal incision. It allows good exposure, may be easily extended for a neck dissection when necessary, and hides unsightly scars in natural skin creases and hairlines, giving an acceptable cosmetic result [28]. In every case, the facial nerve should be protected by using intraoperative facial nerve monitoring to detect and follow facial nerve branches, preventing unintended injury [8, 28]. Uneventful postoperative recovery should be expected in every case.

In conclusion, buccal space masses are rare and at the same time quite diverse, making their diagnostic approach very challenging. Whenever surgical management is decided, it may be successfully performed as long as adequate respect to the anatomy is shown, with careful identification and preservation of structures such as the facial nerve branches and parotid duct.
